# Hepatoprotective Effects of Chinese Medicinal Herbs: A Focus on Anti-Inflammatory and Anti-Oxidative Activities

**DOI:** 10.3390/ijms17040465

**Published:** 2016-03-29

**Authors:** Puiyan Lam, Fan Cheung, Hor Yue Tan, Ning Wang, Man Fung Yuen, Yibin Feng

**Affiliations:** 1 School of Chinese Medicine, The University of Hong Kong, Hong Kong, China; fifilam@hku.hk (P.L.); cheungfan@connect.hku.hk (F.C.); hoeytan@connect.hku.hk (H.Y.T.); ckwang@hku.hk (N.W.); 2 Division of Gastroenterology and Hepatology, Queen Mary Hospital and Department of Medicine, Li Ka Shing Faculty of Medicine, The University of Hong Kong, Hong Kong, China; mfyuen@hku.hk

**Keywords:** liver diseases, Chinese medicinal herbs, anti-inflammatory, anti-oxidative, hepatoprotection

## Abstract

The liver is intimately connected to inflammation, which is the innate defense system of the body for removing harmful stimuli and participates in the hepatic wound-healing response. Sustained inflammation and the corresponding regenerative wound-healing response can induce the development of fibrosis, cirrhosis and eventually hepatocellular carcinoma. Oxidative stress is associated with the activation of inflammatory pathways, while chronic inflammation is found associated with some human cancers. Inflammation and cancer may be connected by the effect of the inflammation-fibrosis-cancer (IFC) axis. Chinese medicinal herbs display abilities in protecting the liver compared to conventional therapies, as many herbal medicines have been shown as effective anti-inflammatory and anti-oxidative agents. We review the relationship between oxidative stress and inflammation, the development of hepatic diseases, and the hepatoprotective effects of Chinese medicinal herbs via anti-inflammatory and anti-oxidative mechanisms. Moreover, several Chinese medicinal herbs and composite formulae, which have been commonly used for preventing and treating hepatic diseases, including *Andrographis Herba, Glycyrrhizae Radix et Rhizoma*, Ginseng Radix et Rhizoma, Lycii Fructus, Coptidis Rhizoma, curcumin, xiao-cha-hu-tang and shi-quan-da-bu-tang, were selected for reviewing their hepatoprotective effects with focus on their anti-oxidative and ant-inflammatory activities. This review aims to provide new insight into how Chinese medicinal herbs work in therapeutic strategies for liver diseases.

## 1. Introduction

More than 10% of the world population is affected by chronic liver diseases [1], which may consequently develop to cirrhosis and hepatocellular carcinoma (HCC) caused by progressive destruction and regeneration of liver parenchyma. Liver is not only an important digestive organ, but also closely connected to inflammation, which is the innate defense system of the body for removing harmful stimulus. Liver inflammation caused by infections arise from either exogenous agents such as environmental toxins, or exposure to endogenous reactive oxygen species (ROS). Sustained inflammation and wound regeneration processes in response to chronic liver injury can induce the development of fibrosis, cirrhosis and eventually HCC. Actually, approximately 80% of HCC patients progressed from hepatic fibrosis or cirrhosis [2], and this demonstrates the importance of chronic wound-healing process to hepatocarcinogenesis, where inflammation is a key promoter.

Various inflammatory mediators have been proven their roles of acting as targets or activators of nuclear factor-κB (NF-κB) [3,4,5,6,7,8]. NF-κB is involved in regulating inflammatory signaling pathways and responses in the liver [9,10,11]. Moreover, oxidative stress (OS) is related to the activation of inflammatory pathways [12], and occurs when there is disequilibrium between the production of oxidants or reactive oxygen species (ROS), which are also known as free radicals and reactive metabolites, and this can be relieved by antioxidants. Somatic mutations and neoplastic transformation could be induced by ROS. OS is related to the initiation of cancer and its pathogenesis, via promoting genome instability, cell proliferation, and DNA damage or mutations [13].

Chronic inflammation, caused by chemical, biological and physical factors, is found to be related to certain human cancers [14]. The effect of the inflammation-fibrosis-cancer (IFC) axis acts as a bridge from inflammation to cancer [15], and therefore promotes inflamed liver evolving to fibrosis/cirrhosis and HCC.

The therapy for hepatic diseases has been extensively explored with remarkable progress in the last few decades, however, the outcomes are still not desirable, mostly due to complications incurred and relatively high cost [16]. There is therefore an imminent need for the development of new prophylactic and therapeutic agents.

Traditional Chinese medicine (TCM) has long been used to prevent and treat hepatic diseases since ancient China, and has received more attention from the public in recent years due to its steady supply, long-lasting curative effects and mild complications. Chinese medicinal herbs (CMHs) exhibit hepatoprotective effects via mechanisms including blocking fibrogenesis, suppressing tumorigenesis, eliminating viruses, and inhibiting oxidative injury [17,18].

Considering that OS and inflammation are triggers in the pathogenesis of liver diseases, CMHs show its benefits in hepatoprotection compared to conventional therapy, as many herbal medicines have been shown to be effective anti-inflammatories and anti-oxidative agents.

Therefore, a timely and prospective review related to the hepatoprotective effects of CMHs is needed. Several relevant reviews have been published, for example, Wang *et al.* reviewed the potential prophylactic and curative effects of Chinese medicines on human HCC and its possible mechanisms [19]; Hong *et al.* reviewed the potential role, pharmacological studies and molecular mechanisms of medicinal herbs [20]; Hu *et al.* reviewed anti-HCC compounds derived from Chinese medicine and its pharmacological mechanisms [21]. These reviews mainly focused on the compounds derived from CMHs and the associated pharmacological mechanisms in particular diseases. Here we review how OS and inflammation are related to the pathogenesis of liver diseases, and the hepatoprotective effects of CMHs with a focus on their anti-inflammation and anti-oxidation properties. 

## 2. The Characteristics of Inflammation and Oxidative Stress in Hepatic Disease

Hepatic injury is mostly due to the sustained exposure of the liver to certain substances, like alcohol, viruses, parasites, toxic substances and biotransformed metabolites, and can result in the degeneration and inflammation of the liver, leading to chronic liver diseases (CLDs), which may further progress to different stages of HCC, fibrosis and cirrhosis [22]. Fibrosis is a wound healing process and initiated by inflammation and OS [23,24,25,26], and can finally develop into HCC [27]. OS leads to architectural disarray, destruction of hepatocytes, and focal or zonal necrosis, by inflammation [22].

Alcoholic liver disease (ALD), nonalcoholic fatty liver disease (NAFLD), and chronic viral hepatitis (B and C) are the main causes of liver cirrhosis [25]. The development of chronic hepatic disease is associated with activation of the immune system, recruitment of lymphocytes from the sinusoids vein, hepatic vein and portal tract, upregulation of inducible nitric oxide synthase (iNOS), and infiltration of polymorphonuclear leukocytes (PMN). Liver diseases may also induce hepatopulmonary and hepatorenal syndromes as well as portopulmonary hypertension in some cases [28].

### 2.1. Oxidative Stress (OS) and Reactive Oxygen Species (ROS)

OS is the common etiological factor in most liver diseases, including those induced by ionizing radiation, toxins, drugs, and other chemicals as well as NAFLD, and ALD [29]. Moreover, in the development of many diseases, such as cardiovascular diseases (CVD), chronic kidney disease (CKD), diabetes, obesity and sepsis, the liver could also be injured [30].

ROS is the chemical species with unpaired electrons, which are also known as free radicals, or molecular oxygen derived ions, like hydroxyl radical (HO), singlet oxygen (O_2_), hydrogen peroxide (H_2_O_2_), and superoxide anion radical (O^2−^) [31]. ROS are likely produced from the cytochrome P450 and mitochondria in the hepatocyte, neutrophils and Kupffer cells (KCs) [32]. ROS stimulates neutrophil chemotaxis and form Mallory corpuscles, by crosslinking cytokeratins, and activating transcription factors (activator protein 1 (AP-1), NF-κB, and c-Jun N-terminal kinase (JNK)) to up-regulate the genes implicated in fibrogenesis TIMP metallopeptidase inhibitor 1 (TIMP1), monocyte chemoattractant protein 1 (MCP-1), and pro-collagen type I [26].

The liver is the main organ responsible for detoxification, including clearing pathogens, toxic chemicals, and metabolic waste products, and is involved in maintaining homeostasis [14,22,33]. It contains rich populations of various resident innate immune cells such as dendritic cells, KCs, natural killer (NK) cells and natural killer T (NKT) cells, all of which are associated with liver pathologies [34]. In physiological scenarios, the pro-oxidants like reactive nitrogen species (RNS) reactive nitrogen species, and ROS produced by liver in aerobic metabolism can be sequestrated by antioxidants [35]. ROS acts as vital cellular mediators in different signaling and metabolic pathways [36,37]. However, when it comes to hepatic injury, OS happens, and gives rise to an imbalance between oxidants and antioxidants, thus increasing the generation of ROS [32,38].

Nicotinamide adenine dinucleotide phosphate-oxidase (NADPH) promotes generation of ROS in hepatocytes that leads to DNA damage and apoptosis, in which genes further promote the synthesis of pro-inflammatory cytokines, and eventually initiates the transformation of malignant cells [39]. Also, malondialdehyde (MDA) promotes inflammation via activating NF-κB and 4-hydroxynonenal (4-HNE), which are tissue inhibitors of TIMP1, and responsible for upregulating procollagen and profibrotic stimulus.

The lipid solubility, half-life and chemical reactivity of ROS varies among different species. The ROS with short half-lives can result in the characteristics of high toxicity and reactivity, but limited diffusion. On the contrary, the aldehydic products, like 4-hydroxynonenal (4-HNE) and MDA, can diffuse to other locations intracellularly and extracellularly, and thus increase the activities of OS, since they have longer half-lives [20]. Such products are produced from lipid peroxidation of organelles and cell membranes, resulting from the damage to polyunsaturated fatty acids (PUFAs) by ROS [40,41,42]. Liver may clear the ROS and RNS by enzymes like thioredoxin, catalase (CAT), superoxide dismutase (SOD) and peroxidase (GPx), as well as antioxidants, for instance glutathione (GSH) and vitamins A, C, and E [43,44]. Among those enzymes, SOD is implicated in the transformation of H_2_O_2_ to free oxygen and water by GPx/CAT, the dismutation of O^2−^ to H_2_O_2_ [45].

### 2.2. Leukocytes and Kupffer Cells (KCs)

KCs, also known as Browicz–Kupffer cells and stellate macrophages, release ROS, which is the cause of fibrosis and cirrhosis, through stepping up the synthesis and proliferation of extracellular matrix (ECM), and activating the hepatic stellate cells (HSCs) [26]. The activated hepatic phagocytes are the main sources of OS in liver diseases and one of the resident innate immune cell populations and the main sources of OS in liver diseases [22,34,46]. They involve in all chronic inflammatory liver diseases and tissue response to OS [32,46,47].

Activated KCs release cytokines and inflammatory mediators, such as iNOS by NF-κB mediated mechanism, and interleukins Tumor necrosis factor-α (TNF-α), Interleukin (IL)-1β, IL-6, IL-12, IL-18. They also activate the generation of oxidants, which are involved in bacteria endocytosis, and superoxide induced from NADPH [32,34,48]. The iNOS boosts NO production, thus increasing hepatocyte toxicity and activating particular intracellular pathways, like pro-apoptotic signals through the caspase cascade [49,50].

Apoptosis destroys certain amount of hepatocytes and this initiates the vicious cycle of liver damage, as the injured hepatocytes not only jeopardize the liver function, but also activate KCs and let out the apoptotic bodies, contributing to inflammation and fibrogenic responses [49]. The activation of KCs exacerbates inflammation through gathering neutrophils and mast cells, and accumulates platelets that hinder local microcirculation, therefore causing ischemia reperfusion [49]. Moreover, the mast cells and leukocytes recruited for the inflammation further deteriorates the situation, in which more oxygen has to be consumed, and worsens the cellular respiration, thus stimulating the production and accumulation of ROS [51]. Furthermore, morphological and functional changes due to the inflammatory mediators, ROS, depletion of antioxidants, mitochondria damage, inflammatory mediators, and overexpression of pro-apoptotic proteins, can provoke acute inflammatory response, therefore causing some complications, such as fibrosis and cirrhosis [49,50]. Fibrosis is the accumulation of too much ECM, and progresses to cirrhosis, which is accompanied with the portal hypertension, damage to normal liver structure, development of nodules and septae, and the evolution to hepatic insufficiency and HCC [21,52].

### 2.3. Hepatic Stellate Cells (HSCs)

The activated HSCs, also previously known as vitamin A-rich cells, fat-storing cells, perisinusoidal cells, Ito cells, or lipocytes, are related to the formation of ECM components (e.g., collagen types 1,3,4), the alterations in cellular functions and increased smooth muscle α-actin (αSMA) expression, which in turn promotes subpopulations of stellate cells [53,54,55]. The activation of HSCs results in inflammation via promoting the release of pro-inflammatory cytokines that provokes apoptosis, fibrogenesis, and hepatocyte necrosis [55]. The deactivation of hepatic stellate cells promote the completion of fibrogenesis and regression of the extracellular matrix [49]. Proliferation, fibrogenesis, and contractility of HSC could be altered by perpetuation phase, which is created when injuries are under continuous stimuli and maintenance, as well as regulated by autocrine and paracrine stimulation [56].

The damage to hepatocytes, as well as those chemokines and cytokines derived from KCs, e.g., platelet-derived growth factor (PDGF), TNF-α, IL-1, and tissue growth factor (TGF)-β1, can help the transformation of the activated hepatic HSCs to myofibroblasts, and therefore induce hepatic fibrogenesis [49]. Moreover, the damaged hepatocytes release mediators, like ROS/RNS, MDA/4-HNE, cytokines, and hepatotoxins, which are associated with the activation of HSC [49].

### 2.4. Oxidative Stress and DNA Methylation

OS and inflammation are major parts in the development of hepatic diseases, of which DNA methylation is possibly the pivot point. Some studies have shown that the physiologic and pathologic activities are involved in ROS and DNA methylation reactions [57]. DNA methylation is a postreplication epigenetic modification and the methylation of cytosine-phosphate-guanine (CpG) dinucleotide cytosines [58], leads to 5-methylcytosine. Methylated cytosines in human somatic cells [59], unsymmetrically shown in the genome with CpG-rich or -poor regions, influence 70%–80% of all CpG dinucleotides and cover 1% of total DNA bases. The methylated cytosines represented in CpG-rich regions, also known as CpG islands, are usually nonmethylated in normal cells [60] and include promoter regions including the first exons of certain genes [61]. DNA methyltransferases (DNMTs) participate in DNA methylation, of which DNMT1, DNMT3a and DNMT3b are enzymes responsible for the methyl group to be transferred from *S*-adenosylmethionine (SAM) to cytosine. DNMT1 is found in somatic cells and involved in maintaining DNA methylation, by copying methylation patterns to DNA strand after replication [62,63], which is essential for chromosome X inactivation, proper embryo development and heredity [64,65]. DNMT1 deletion impairs the monoallelic expression of various hereditary genes, which is based on the parental root of the allele. DNMT1 expression increased after melanocyte anchorage blockade [66] and global DNA hypermethylation resulted after elevation of superoxide anions. DNMT3a and DNMT3b are also required for *de novo* methylation in the genome after embryo implantation and development [67,68,69,70,71]. Investigators pointed out that these three enzymes are involved in *de novo* methylation maintenance and pattern [72,73]. DNA methylation inhibits gene transcription by the location and density of the promoter CpG islands [74,75,76].

Demethylating agents demonstrate the effect of DNA methylation in stable gene inactivation, such as reducing the inactivation of retroviruses [77] and chromosome X [78,79,80]. This inactivation activity was demonstrated by reactivating somatic cells in culture and X transgenes of mouse embryo with inhibited or deficient DNMT1 [81]. As epigenetic modifications suppress one of the two alleles for the same cell, DNA methylation is involved in heredity, however the other alleles remain active [81]. On the whole, DNA methylation is the important process, in which DNMT1 preferentially suppresses a copy of a gene during cell division based on the parental origin [68,82].

Moreover, gene footprints are found epigenetically deregulated in various pathologies and human syndromes [77]. For example, low expression of *SOD* in born preterm adults [83] and OS are related to DNA hypermethylation of a single CpG dinucleotide. Epigenetic mechanisms speed up DNA reacting to the positive charged intermediate SAM [66], by the influence from ROS overproduction [84,85], where superoxide anions deprotonate the cytosine molecule and act as nucleophilic agents. Besides, the elevation of methylation of RUNX3 (runt-related transcription factor 3) in cells exposed to H_2_O_2_ [86], which is an epigenetic mechanism controlling *SOD2* transcriptional activity throughout the pathogenesis of human cancers [87], and upregulation of DNMT1 in colon cancer-derived cell lines [88], result in H_2_O_2_-mediated epigenetic modifications. It was also observed from rat fetal hearts that norepinephrine-induced ROS production reacted to increased DNA methylation of the protein kinase C promoter [89].

In contrary, a vicious cycle established by decreased SOD activity may result in altered epigenetic regulation, hence further stimulates epigenetic instability [82]. Several studies have demonstrated that the antioxidant defenses are impaired by DNA methylation in cancers. For instance, the *SOD2* promoter is hypermethylated in peripheral blood mononuclear cells [90,91], while the promoter of extracellular SOD is strongly hypomethylated in fibroblasts of human embryonic lung (MRC5) [92].

In addition, hyper methylation appeared in various isoforms of glutathione peroxidase, such as GPx7 and GPx3, in cancers and this could be repaired by 5-aza-2’-deoxycytidine [93,94,95,96]. The double-knockout mice model with intestinal cancer showed that the aberrant methylation of polycomb target genes mediated by inflammation are deficient in both *GPx1* and *GPx2* [94]. Moreover, from the mice model with prostate cancer, it is observed that erythroid 2-related factor 2 (Nrf2), a transcription factor responsible for the gene activation of antioxidant enzymes, was hypermethylated, whereas it was re-expressed by curcumin with hypomethylating effect [97]. It is also shown that low gene and protein expression appears [98], when catalase is hypermethylated in the promoter CpG II island after long time exposure to ROS.

In general, DNA methylation, is able to damage the expression of antioxidant genes like *GPx* and *SOD*, hence exacerbating OS and inflammation in hepatic diseases. Propitiously, many natural agents, including curcumin [97], polyphenols (e.g., epigallocatechin 3-gallate from soya genistein and tea) [99,100,101], selenite, and methyl donor substrates for DNMTs (vitamin B, methionine and folates) have the competency to inhibit or reverse these events, most of which could be found in CMHs.

## 3. Inflammation and Oxidative Stress Properties in Major Hepatic Diseases

### 3.1. Hepatocelullar Carcinoma (HCC)

HCC is the major type of primary liver cancer [102], and mostly associated with patients with HBV, hepatitis C virus (HCV), excessive alcohol consumption and NASH [102,103,104]. OS and inflammation both contribute to its pathogenesis [40], where OS is associated with the progress of HCC via increasing the malignant characteristics of HCC and telomere shortening in hepatocytes. OS participates in some intracellular signaling cascades like oxidation of DNA which has mutagenic effect in mammalian cells [41], and cell signaling, especially transcription factors like NF-κB and AP-1, as well as expressions of cytokines like TNF-α and IL-β1. Moreover, OS regulates matrix metalloprotease 1 (MMP1). Consequently, apoptosis is increased and results in carcinogenesis via the generation of ROS stimulated by increased OS [42].

Moreover, inflammation is involved in HCC carcinogenesis, in which activation of NF-κB stimulates generation of pro-inflammatory cytokines including cyclooxygenase (COX)-1, COX-2, TNF-α, C-reactive protein (CRP), IL-1, IL-26, IL-8, IL-18, macrophage inflammation protein (MIP)-1α, and 5-LO [105]. Those pro-inflammatory cytokines promote the hepatic and systematic inflammation, which then changes the microenvironment in the liver and leads to fibrosis and abnormal hepatocytic regeneration [106].

### 3.2. Hepatitis C Virus (HCV)

HCV is a chronic hepatic disease with high-incidence rate and it reached 185 million infections worldwide in past 15 years [107]. It can develop to cirrhosis and HCC, which is accounted for 23% of HCV patients [108] due to the sustained cellular damage. The inflammation is mainly responsible for the pathogenesis of HCV, and is closely associated with OS, as well as the development of liver fibrosis and cirrhosis [109]. It is observed in all types of liver injuries that the increase of ROS production is related to the decrease of antioxidant defense [110,111]. Hence, OS is implicated in the pathogenesis of HCV, HCC and other liver diseases [112,113]. Endoplasmic stress (ER), resulting from HCV gene expression, decreases the ER calcium accumulated and increases calcium uptake in the mitochondria, thus promoting ROS generation which changes the nature of proteins via lipid peroxidation [114]. Activator of transcription 3 (STAT-3) and NF-κB are then activated by ROS, via the activation of serine/threonine kinases and cellular tyrosine. NF-κB is transported into the nucleus, thus activating the pro-oxidant and pro-inflammatory genes [115].

Iron overload is another way that OS is involved in HCV infection [116]. It was observed that there is a significant surge of Fe^+3^ in the liver and serum of the HCV patients [117,118], though the mechanism is unclear. The accumulation of hepatic iron stimulates the generation of HO^−^, which causes liver injury via reacting with lipid membranes, proteins and DNA [119].

Moreover, the redox imbalance of HCV patients also dampens the endogenous antioxidant defense [120]. Vitamin A deficiency is associated with HCV patients and this leads to poor responsiveness to interferon-based antiviral treatment [121], as more than 90% of vitamin A in the body is stored in the liver and serves as the main exogenous antioxidant. Also, the HSCs activated by ROS, cause hepatic fibrosis in HCV patients. Redox changes can also be observed in the decrease of total antioxidant status (TAS) and SOD, as well as the increase of MDA levels.

### 3.3. Hepatitis B Virus (HBV)

It is estimated that around 30% of population in the world are infected with HBV and it can cause liver fibrosis, HCC, hepatic complications and other serious illness [122,123,124]. Metabolism of the host cells changes due to the HBV infection, and this in turn promotes its replication and expression, which promotes hexosamine and phosphatidylcholine biosynthesis, as well as upregulation of genes related to lipid biosynthesis, and glutamate dehydrogenase 1 and isocitrate dehydrogenase [125,126,127]. Participation of OS in the pathogenesis of HBV can be visualized by increase of MDA, decrease of GPx activity, OS index (OSI), total oxidant status (TOS), TAS, carbonyl levels, GSH consumption, β-carotene, and ceruloplasmin levels [128,129]. Besides, OS is involved in viral replication via four open reading frames, including Hepatitis B virus x (HBx) protein, Pol (polymerase), Sp (surface protein), and denoted Cp (core protein), which are related to the elevation of H_2_O_2_ and elevation of GSH levels, and involved in the double-stranded DNA genome of HBV [130]. Hence, OS appears to have a great impact on DNA damage and hepatocarcinogenesis.

### 3.4. Alcoholic Liver Disease (ALD)

**Figure 1:**
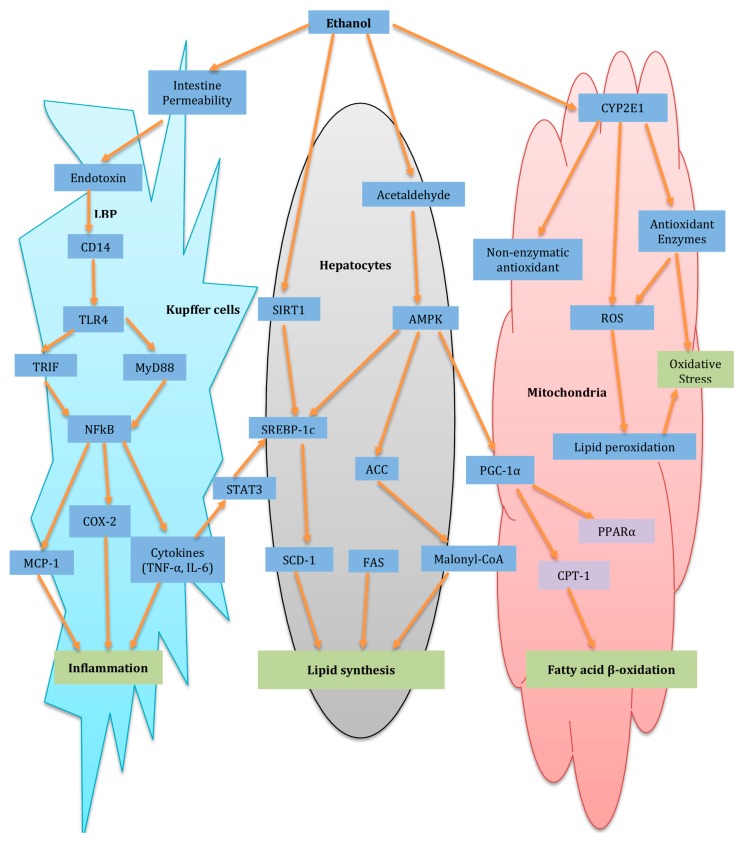
Schematic diagram of major pathways of alcoholic fatty liver (ALD) and potential molecular targets of herbal medicine for the protection of ALD. The arrows indicate the potential molecular targets involved in the development of ALD and regulated by herbal medicines. ACC: Acetyl-CoA carboxylase; AMPK: AMP-activated protein kinase; CD14: cluster of differentiation 14 COX-2: Cyclooxygenase-2; CPT-1: Carnitine palmitoyltransferase-1; CYP2E1: Cytochrome P450 2E; FAS: Fatty acid synthase; IL-6: Interleukin 6; MCP-1: Monocyte chemotactic protein-1; MyD88: Myeloid differentiation factor 88; NF-κB: Nuclear factor-κB; PGC-1α: Peroxisome proliferator-activated receptor g coactivator α; PPARα: Peroxisome proliferator activated receptor RNS Reactive nitrogen species; ROS: Reactive oxygen species; SCD-1: Stearyl CoA desaturase-1; SIRT1: Sirtuin 1; SREBP-1c: Sterol regulatory element-binding protein-1c; STAT-3: signal transducer and activator of transcription-3; TLR: Toll-like receptor 4; TRIF: TIR-domain-containing adapter-inducing interferon-b; TNF-α: Tumor necrosis factor-α.

As one of the most prevalent hepatic injuries in the world, ALD accounts for 4.6% and 3.8% of all disability and mortality adjusted life-years, respectively, incurred from ethanol consumption [131]. It can evolve into different severity, from steatosis and even to cirrhosis [132]. ALD can be histologically classified into three stages: fatty liver/hepatic steatosis, alcoholic hepatitis and chronic hepatitis with hepatic fibrosis/cirrhosis, with inflammation and OS involved in different stages [133]. Actually, emerging evidence demonstrated that there are multiple mechanisms involved in ALD, including not only OS and inflammation, but also complex interactions between the immune system, lipid metabolism and alcohol metabolism, as well as excess lipid synthesis [134]. The schematic diagram of major pathways of alcoholic fatty liver (ALD) and potential molecular targets of herbal medicine for the protection of ALD is summarized in Figure 1.

#### 3.4.1. Oxidative Stress and Inflammation in Pathogenesis of ALD

The significance of ethanol-mediated OS in the pathogenesis of ALD was revealed [135,136]. Alcohol consumption, whether chronic or acute, suppresses cellular antioxidant levels, which leads to OS in various tissues, mainly in the liver, and boosts the generation of ROS, including hydrogen peroxide, superoxide, and hydroxyl radical [137]. As alcohol-induced ROS could enhance lipid peroxidation of cellular membrane and cause DNA damage, that inhibits physiological activities and promotes OS, via reaction with most cellular macromolecules by inactivating enzymes or denaturing proteins, thus its production is toxic to hepatocytes [137].

As a form of cytochrome P450 enzyme, Cytochrome P450 2E (CYP2E1) has been thought to contribute to ROS production in response to alcohol consumption. Its activity and expression are increased by alcohol intake, which catalyzes the ethanol process to acetaldehyde in the presence of iron, thus leading to overproduced ROS [138]. A number of compounds derived from CMHs, like methanol extract from the roots of *Platycodon grandifloras* (Jacq.) A.DC. [139] and *Gentiana manshurica* Kitag. (Gentianaceae) [140], have been demonstrated to inhibit CYP2E1 catalytic process *in vitro* or reduce CYP2E1 expression that causes attenuation of lipid peroxidation and ROS generation.

Endogenous antioxidant enzyme systems act as a first defense against oxidative damage and are associated with ROS elimination, including superoxide dismutase (SOD), glutathione peroxidase (GPX), glutathione-*S*-transferase (GST) and catalase (CAT) [137]. However, alcohol consumption, especially chronic, could damage enzymatic and non-enzymatic antioxidant systems that protect hepatocytes from ROS damage [137].

SOD is responsible for keeping cellular redox balance and scavenging ROS, which is crucial for endogenous anti-oxidative defense system, whereas GPX catalyzes the decrease of hydrogen peroxide and other peroxides [141].

Non-enzymatic antioxidants, like vitamins C and E, and the reduced form of glutathione (GSH), participate in keeping the cell safe from lipid peroxidation. GSH, the most plentiful tripeptide thiol antioxidant, acts as the substrate of GSH-related detoxifying enzymes and antioxidants, as well as a direct ROS scavenger [141].

Hence, increasing these antioxidants may be beneficial in eliminating OS and removing ROS induced by alcohol intake. Moreover, alcohol consumption can deplete endogenous vitamins C and E, which are the non-enzymatic antioxidants and can be resorted by leaf water extract from *Cassia auriculata* [142] and fenugreek seed polyphenol [143].

Lipid peroxidation (LPO) is the process of oxidative degradation of lipids, in which free radicals are produced by ethanol and its metabolites [144]. Malonyldialdehyde (MDA), an end-product of LPO, has been broadly adopted as an index for the status of OS and LPO [144]. Various studies have indicated that different extracts from CMHs, for instance Ginkgo biloba extract [141,145], and curcumin [146], could reverse the increase of hepatic MDA level resulting from chronic alcohol ingestion.

The bacterial lipopolysaccharide (LPS, endotoxin) is transferred into the portal circulation and then into the liver by the effect of alcohol intake, where LPS could activate Kupffer cells and trigger a liver inflammatory injury, via interfering with the epithelial barrier and hence increasing intestinal permeability to macromolecules [147,148]. The intestinal barrier function is proven to be a key biological barrier against the toxic dietary and luminal substances, by protecting the penetration of luminal antigens [149]. Ethanol extract of *Pueraria lobata* has showed its significant ability to curb ALD via suppression of ethanol induced-increase of intestinal permeability [149].

Kupffer cells also participate in hepatic inflammation in ALD [134,150]. Gut-derived endotoxin, the protein complex adhering to LPS-binding protein (LBP) after getting into portal circulation, is identified by Toll-like receptor-4 (TLR4) and its co-receptors, like cluster of differentiation 14 (CD14), on the cell membrane of Kupffer cells, that triggers inflammation by further activating Kupffer cells and the downstream TLR4-mediated pathways [150,151].

TLR4 activates pathways via recruiting adapter molecules, consisting of TIR-domain-containing adapter-inducing interferon-b (TRIF) and myeloid differentiation factor 88 (MyD88), which activates NF-κB and subsequently discharges various inflammatory mediators [150,152].

It was observed that individual compounds and crude extracts isolated from different CMHs could suppress the activation of Kupffer cells via interfering with TLR4 pathway. In particular, Baicalin from ethanol extract from Cinnamomi Cassiae Cortex [153], *Scutellaria baicalensis* Georgi (Lamiaceae) [154], and aqueous extract of *Agrimonia eupatoria* L. (Rosaceae). [152] suppressed nuclear translocation of NF-κB via suppressing the expression of MyD88 and TLR4. Besides, curcumin from *Curcuma longa* L. (Zingiberaceae) [146,155] has been demonstrated to curb NF-κB activation in liver.

Moreover, NF-κB, a key transcription factor modulating the transcription of many inflammatory genes in ALD, causes increased expression of inflammatory factors, which include eicosanoid metabolism enzymes (e.g., cyclooxygenase-2, COX-2) that synthesize inflammatory lipid mediators chemokines (e.g., MCP-1), adhesion molecules, and cytokines (e.g., TNF-α, IL-1, IL-6, IL-8 and IL-12) [156]. TNF-α, a cytokine produced by sensitized Kupffer cells and recruited monocytes, has been broadly thought to be vital to alcohol-induced hepatic damage, other than direct toxic effect on hepatocytes. TNF-α possibly ameliorates fatty acid *de novo* synthesis [157], for instance, curcumin from *Curcuma longa* also inhibits expression of cytokines COX-2 in isolated Kupffer cells, chemokine (MCP-1) and (TNF-α and IL-12), and stops LPS-mediated activation of NF-κB [155].

#### 3.4.2. Lipid Synthesis and Fatty Acid β-Oxidation in Pathogenesis of ALD

Hepatic steatosis often occurs in chronic alcohol consumption. Hepatic steatosis is due to triacylglycerols (TG) accumulation in hepatocytes, and it was shown in some studies that the development of ALD during alcohol consumption is slowed down by reducing fat accumulation in liver [158,159]. Increased lipogenesis is closely related to hepatic TG accumulation, and causes excessive *de novo* fatty acids and TG synthesis in hepatocytes. It is believed that a transcription factor sterol regulatory element-binding protein-1c (SREBP-1c) is pivotal in regulating lipid homeostasis via moderating the expression of more than 30 lipogenic genes [140,160]. Studies showed that administration of some herbal extracts and single compounds, such as methanol extract from *Gentiana manshurica* [140], ethanol extract from *Magnolia officinalis* [161], and Green Tea extract [162], honokiol [161], resveratrol [163], and caffeine [134], can restrain increased maturation of SREBP-1c in the liver caused by alcohol consumption. SREBP-1c-regulated lipogenic enzymes participates in TG synthesis, such as diacylglycerol acyltransferase (DGAT) [140,160], and fatty acid synthesis, for instance ATP-citrate lyase (ACLY), acetyl-CoA carboxylase (ACC), fatty acid synthase (FAS) and stearyl CoA desaturase-1 (SCD-1). ACLY and ACC are enzymes involves in the carboxylation of acetyl-CoA and synthesis of cytosolic acetyl-CoA to produce malonyl-CoA, respectively. FAS is responsible for synthesizing the long-chain fatty acids from acetyl-CoA and malonyl-CoA, which are unsaturated by SCD-1 [161,164]. The increased expression of lipogenic enzymes due to alcohol ingestion could be inhibited by various crude extracts and individual compounds isolated from CMHs, such as resveratrol [163], honokiol and ethanol extract from *Magnolia officinalis* [161,164].

Moreover, alcohol-induced SREBP-1c activation might be modulated by curbing sirtuin 1 (SIRT1) activity [158] and AMP-activated protein kinase (AMPK) activity [158,165]. AMPK has been reported as a key regulator of lipid metabolism via modulating SREBP-1c activity by decreasing its protein levels and mRNA, and its downstream lipogenic genes in hepatocytes [165]. In the development of alcoholic liver inflammation, TNF-α and IL-6 might not only participate in upregulating the SREBP-1c activity, through activation of signal transducer and activator of transcription-3 (STAT-3) [166,167], but also through contributing to lipid synthesis.

Carnitine palmitoyltransferase-1 (CPT-1) is vital for regulating the transportation of fatty acids from cytoplasm into mitochondria where fatty acids are metabolized through mitochondrial β-oxidation pathway [158]. AMPK-induced inhibition of ACC causes alleviated synthesis and elevated degradation of malonyl-CoA and hence the alleviated malonyl-CoA inhibiting mitochondrial CPT-1, thus causing increased influx of fatty acids into mitochondria and subsequent oxidation. Peroxisome proliferator-activated receptor coactivator α (PGC-1α), a transcriptional coactivator, stimulates target gene transcription implicated in mitochondrial fatty acid utilization and oxidation via interacting with PPARα [168]. Moreover, alcohol consumption inhibits mitochondrial fatty acids oxidation and *CPT-1* gene expression, which might cause fatty acid overload and hepatic fat accumulation [169].

### 3.5. Non-Alcoholic Steatohepatitis (NASH)

NASH is prevalent in chronic liver diseases, and found in 20%–30% in the general population with this condition, of which 70%–90% are patients with obesity and diabetes [170]. Actually, it is an extreme case of non-alcoholic fatty liver disease (NAFLD), which may lead to fatty liver where fat is deposited in the liver resulting from causes other than alcohol consumption. NASH causes inflammation and hepatic cell damage, thus finally developing into cirrhosis and HCC. Many factors are associated with the pathogenesis of NASH, such as insulin resistance (IR), inflammation, OS, advanced glycation end products (AGEs), and lipid metabolism alterations [171].

Day *et al.* suggested the “two-hit” theory to explain the participation of inflammation and OS in NASH [172]. The first hit is the accumulation of free fatty acids (FFA) and triglycerides (TG) into hepatocytes, via increasing IR, dietary influx, and hepatic lipogenesis; the second hit is hepatocyte damage and progression of liver fibrosis by lipid peroxidation, inflammation and mitochondrial dysfunction [171].

**Figure 2:**
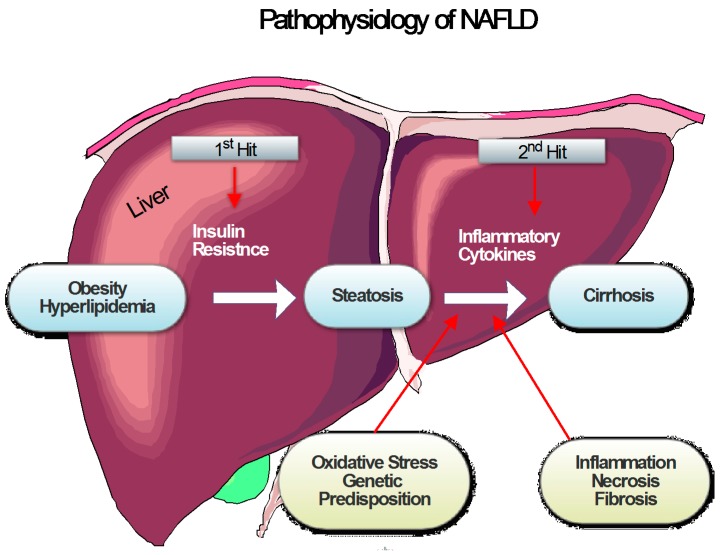
Pathophysiology of nonalcoholic fatty liver disease (NAFLD). The pathogenesis can be explained by the “two hit” hypothesis, and the different grades of severity are indicated by the white arrow. Various factors are involved in the development of NAFLD and represented by the red arrows.

Nitrogen species (RONS) is associated with regulating lipid metabolism through the activation and inhibition of signal pathways, such as the pathogenesis of steatohepatitis due to immune system activation and enhancement of adipokines. Moreover, signal pathways induced by RONS may initiate IR [173]. Changes of markers of redox and inflammation also can be observed from NAFLD/NASH patients, for instance AGEs, increased high sensitivity C-reactive protein (hsPCR), of which the MDA and hydroxyl radical-mediated oxidation of lipids, as well as the decrease of TAS and SOD are involved [172,174,175,176,177,178]. NASH-associated inflammation may be due to gut microbiota, as a response to the circulating inflammatory cells, inflamed adipose tissue, as well as infiltration of macrophage-inflammatory protein-2 and neutrophil chemokines. ROS is also involved in hepatic inflammation by means of the activity of cytokine and enzyme CYP2E1, which is associated with the generation of ROS, and increased expression in NASH [179]. The pathophysiology of nonalcoholic fatty liver disease NAFLD is demonstrated in Figure 2.

### 3.6. Drug-Induced Liver Injury (DILI)

Due to different characteristics of drugs, the pathogenic factors of DILI varies and these include OS, interference on mitochondrial respiration, physicochemical characteristics, depletion of antioxidants, reactive metabolites formation, and threshold dose [35,180]. The OS incurred form DILI may be resulted from the cytosolic stress in drug metabolism by injured liver cells [180]. Acetaminophen (APAP) is the most common example of DILI inducer [35,180].

N-acetyl-p-benzoquinone imine (NAPQI) is generated by OS implicated in APAP with cytochrome P450, and is a toxic metabolite, which not only oxidizes the thiol group of GSH to reduce the GSH/ GSSG ratio, but also attacks and modifies proteins covalently [181]. APAP exhibits oxidative capacity and leads to hepatotoxicity via generating peroxidation reaction products and RNS and ROS. The drug toxicity can initiate inflammation via generating cytokines by KCs and neutrophils, e.g., IL-1α, IL-1β, IL-6, IFN-γ and TNF-α [182]. These cytokines control the adaptive immune-mediated cell damage, or promotes hepatocytes to biochemical stress. Furthermore, an antigen, the drug-protein adducts, initiates the adaptive immune activities via binding T-cell receptors of CD4 cells, thus activating CD8 cytotoxic T-cells.

## 4. Current Strategies for Anti-Inflammation and Anti-Oxidation

Corticosteroid is one of the most commonly used medications for the inflammation and can regulate the fat and protein metabolisms. However, corticosteroid therapy has side-effects such as sepsis and gastrointestinal hemorrhage, and this limits its benefit to the patients [183,184]. Comparing to applying corticosteroids alone, the combined interventions of corticosteroids and *N*-acetylcysteine showed fewer incidences of hepatorenal syndrome and infection. But the related infections possibly have to be further treated by antibiotics, as it is considered as the contradiction to the drug treatment [185]. Besides, pentoxifylline is considered as a favorable medication for patients with sepsis complications due to its anti-tumor necrosis factor (anti-TNF) and antioxidant effects, thus alleviating hepatorenal syndrome [183,186,187].

Chemically selective substances used by anticytokine synthesis therapy exhibited obvious effects to inhibit inflammation, but are still not introduced into clinical practice, as cytokines can promote liver regeneration, though their inhibition can restrain hepatic diseases [185,188]. Inhibition of interleukin-receptor (IL-R) showed some side-effects including delay of liver regeneration and increase of bacterial infection [185,188]. IL-22 is another hepatoprotective cytokine and has fewer side effects for treating liver injuries. Its combination with steroid and TNF-α inhibitors may eliminate the steroid-induced bacterial infection and stimulate liver regeneration, due to its antioxidant, antimicrobial and antiapoptotic and antisteatotic effects. And quite to the contrary, anti-TNF-α therapy alone is more prone to cause severe infection and death. Nonetheless, IL-22 may promote hepatic carcinogenesis by promoting cell proliferation and survival of liver tumors. It is only safe to be used for alcoholic hepatitis patients without cirrhosis and hepatic carcinoma [183,188,189,190,191].

COX inhibition is another option of decreasing the production of prostaglandin, and the analgesic effect of aspirin is an example. However, aspirin treatment also has adverse effects, especially in gastrointestinal complications and antiplatelet activity, and therefore nonsteroidal anti-inflammatory drugs (NSAIDs) have been introduced. Ibuprofen, ketoprofen, piroxicam and indomethacin are the most well known NSAIDs, but they also have been observed to present allergy symptoms to patients. Therefore, such COX-2 inhibitors as roficoxib and celecoxib were withdrawn in 2004.

COX-1 (constitutive isoform) and COX-2 (inducible isoform) are two types of cyclooxygenases. NSAIDs were reported to have higher anti-inflammatory effect toward COX-2 than COX-1, and considered as powerful drugs for anti-inflammation, as they have lesser adverse effects by inhibiting COX-2. When inflammation happens, COX-2 is activated to produce pro-inflammatory prostaglandins and thromboxane. It was observed that COX-2 converts free arachidonic acid to prostaglandin precursor, prostaglandin H2, which is then converted to prostaglandin E2 in turn, so as to mediate inflammation. Hence, NSAIDs are introduced to the inhabitation of synthesis of thromboxane and prostaglandin. Likewise, lipoxygenase (LOX) plays a critical role as an inflammation mediator by converting fatty acids into pro-inflammatory leukotrienes, and promotes the production of cytokines to intensify inflammation. Therefore, the anti-inflammatory therapy also targets to the enzyme [192].

The principal function of antioxidants is to antagonize OS so as to prevent or delay the oxidation of substrates, such as lipids, proteins, DNA, DNA mutations, and other cell damage [193]. Vitamin E supplementation is related to an obvious decrease in protein oxidation, lipid peroxidation and increase in the antioxidant defense system. Vitamin E may relieve liver diseases by lowering OS. While early studies pointed out that antioxidant supplementation could be beneficial to health, some current studies report that excess intake (greater than 400 IU/day/vitamin E) of particular supplementations could be harmful and even result in mortality [194,195,196,197,198,199]. It appears that single antioxidant supplementation may not be beneficial, however diets with high antioxidants from fruit and vegetables are good for health. The explanation may be that the mixture of antioxidants from fruits and vegetables presents as a continuous antioxidant chain, while the supplementations mostly work as the combination of not more than two substances [194,195,196,197,198,199,200]. The antioxidants from the incomplete chain cannot be restored and becomes pro-oxidant after scavenging free radicals, and results in non-effective or harmful supplementations [193,201]. Therefore, vegetables, fruits, and herbal drugs with high anti-oxidative effects are more preferable than complimentary antioxidants in antioxidative therapy [193,201], and they can relieve systemic OS [202,203,204,205,206].

As the pathogenesis of liver diseases is associated with OS and inflammation, anti-oxidative and anti-inflammatory therapies should have potential value in its treatment. CMHs have relatively less side effects, and most of them have anti-inflammatory and OS effects [207,208].

## 5. The Anti-Inflammatory and Anti-Oxidative Activities of Herbal Chinese Medicine for Hepatic Diseases

Since most of the CMHs have anti-inflammatory and OS effects with lesser side effects to humans, it could be the new perspective of the therapy to hepatic diseases [207,208]. In this review, some major CMHs and formulae commonly prescribed for treatment and prevention of liver diseases are examined for their anti-oxidative and anti-inflammatory effects.

### 5.1. Epigenetics in Traditional Chinese Medicines for Hepatic Diseases

Epigenetics pertains to gene expression with mitotically stable alterations, of which DNA sequence is not involved. The epigenetic mechanisms in mammalian cells involve RNA interference, post-translational modifications of histone proteins, and CG dinucleotides methylation [209,210]. Gene silencing is associated with microRNA (miRNA) expression, histone deacetylation and DNA methylation, [211,212]. Environment perturbations can easily influence miRNA expression profile and epigenetic marks (epigenome) [213,214]. The predisposition of such diseases as autoimmune disease, Alzheimer’s disease, and heart disease increases with that of an aberrated profile [215,216,217]. Especially, gene-specific hypermethylation and genome-wide hypomethylation are regarded as distinctive features of cancerous cells [218]. The normal development of eukaryotes including plants [59,219] and animals are closely associated with epigenetic events [220].

Actually, four dietary sources (*i.e.*, tea, soy, cabbage and turnip) are believed to be responsible for DNA methylation modification [221]. Tea and turnip are revealed as interacting with MBD, which may interact with DNMTs [222]. Many of the CMHs, 29.8%, have an impact on the miRNA expression and epigenomes of human cells. It was demonstrated that there are 48,491 chemicals among 3294 CMHs which interact with epigenetic-related proteins, 29.8% of which are miRNA- and epigenome-modulating through interactions with methyl CpG-binding proteins and the Polycomb group [223]. Composite formulas are commonly used in TCM clinical practice. Therefore, the role of epigenetics in TCM should be evaluated by examining the participation of epigenetics in composite formulas, which are determined as epigenetic when at least one of the composing herbal medicines is epigenetic. It is demonstrated that though only 30% of the TCMs are epigenetic, 99% of the composite formulas are found to be epigenetic [223]. Moreover, the long-term and holistic effects of TCM prescriptions may be due to their epigenetic characteristics, of which the related proteins are numerous and acquired patterns are mitotically stable.

Over 1500 miRs have been identified in humans and they are now becoming innovative therapeutic agents in multiple diseases, as revealed by their miRNA profiling of particular hepatic diseases such as drug-induced liver injury, ALD, NAFLD, and chronic hepatitis C and B [224,225]. For example, miR-122 has been shown to protect HCV RNA against nucleolytic degradation, increase translation of viral proteins, and promote HCV replication via connecting to various sites in 50 untranslated regions of the HCV RNA genome [226]. In addition, miR-122 regulates insulin, lipid metabolism and iron homeostasis. Antagonizing it may exert numerous positive effects, such as reducing cholesterol levels and low density lipoprotein (LDL) via controlling fatty acid biosynthesis genes and hepatic cholesterol, thus resulting iron deficiency with lower levels of plasma and liver iron as well as β oxidation of fatty acids [227].

Histone deacetylases (HDAC) and histone acetyl transferases (HAT) are two groups of enzymes conducting the acetylation of histones. HDAC3 is related to hepatosteatosis, hepatic energy metabolism and circadian regulation [228]. The evolution of “insulin hypersensitivity’”, triglyceride accumulation and marked steatosis are stimulated by liver-specific knockdown of HDAC3. It keeps normal blood glucose during daytime, and drops it down to stimulate lipid at night, via promoting metabolic sources towards gluconeogenesis and negatively regulating lipogenic genes. Moreover, reduced HDAC3 expression levels are always associated with global acetylation of histones in macrophages and hepatocytes [229].

Hepatic wound-healing and fibrosis are closely associated with the transdifferentiation of hepatic stellate cells (HSC) to a myofibroblast-like phenotype. This modification in phenotype related to HSC transdifferentiation is supported by global alterations in gene expression.

While DNA methylation exists in some genes that are highly expressed in inactivated HSCs, it is silenced as the cells are activated. DNA methylation may be the novel therapeutic target for preventing and treating liver fibrosis. For example, baicalin and rosmarinic acid (active ingredients of composite formulas of TCM, Yang–Gan–Wan), prevent methyl-CpG binding protein 2—enhancer of zeste homolog 2 (MeCP2-EZH2) relay, hence prohibiting hepatic fibrosis via allowing re-expression of PPAR-c [230].

Thus, it is crucial to understand the interactions between methyl binding proteins, DNA methylation and enzymes that control histone modifications, so as to design interventions targeting these pathways. Due to the complexity of the system, the epigenetic mechanisms and their interactions are still not well understood. With greater knowledge, the epigenome may be able to be selectively modulated by applying CMHs intervention to change the course of diseases.

### 5.2. Chinese Medicinal Herbs

#### 5.2.1. *Andrographis Herba*

TCM utilizes the aerial parts or the leaves of *Andrographis Herba*, which carry abundant medicinally useful phytochemicals, particularly glycosides, flavonoids (>20), and diterpene lactones (>20) [231,232].

*Andrographis Herba* is a famous therapeutic herb for hepatic disorders and upper respiratory tract infections [233,234,235,236,237,238]. Its anti-inflammatory and immune-stimulant properties have also been observed [239]. For treating hepatic diseases, it works by alleviating chronic hepatitis B virus infection, and inducing hepatoprotective and hepatostimulating activities [235,238,240,241]. It has also demonstrated outstanding hepatoprotective properties among 58 chemically defined compounds of plant origin and 107 plants [242]. Moreover, the plant extract is believed to contain enormous amounts of phytochemicals to lower the process of lipid peroxidation. The phytochemicals mainly contain phenolic compounds, which is around 5.96 mg/g of leaf extract [242,243]. The plant extract, at concentrations of 50 mg/kg body weight, exhibited hepatoprotective effects in albino Wistar rats by restoring anti-oxidative enzymes [237].

Andrographolide, a labdane diterpenoid that has been isolated from the stem and leaves of *Andrographis Herba*, showed anti-inflammatory properties by reducing the expression of pro-inflammatory mediators [244,245]. It was demonstrated in *in vivo* and *in vitro* experiments that andrographolide reduced the expression of pro-inflammatory proteins in neutrophils by inhibiting the NF-κB signaling pathway [246,247,248,249,250,251,252,253]. NF-κB is believed to regulate genes associated with innate and adaptive immunity. The IC_50_ of andrographolide was found to prevent the activation of NF-κB [248]. Several studies showed that andrographolide could dampen the nitric oxide synthase (iNOS) and COX-2 expression in neutrophils and microglial cells, and TNF production in macrophages, thus reducing the production of nitric oxide and prostaglandin E2 [254,255,256]. Its anti-inflammatory activities have been demonstrated and result from the interference of the andrographolide to protein kinase C-dependent pathway, phosphoinositol-3-kinase (PI3K)/Akt (also known as protein kinase B, PKB) or extracellular signal-regulated kinase (ERK) 1/2 [251].

ROS-induced OS in tissues or cells leads to alcohol-induced liver damage. OS is often examined according to the levels of antioxidant defense enzymes (e.g., glutathione *S*-transferase and catalase, glutathione peroxidase, and superoxide dismutase), thiobarbituric acid reactive substances (TBARS) and lipid peroxides.

The effects of these enzymes are elevated under stress situations against excessive ROS [257,258,259,260]. Various studies reported that *Andrographis Herba* extract could deter enzymes from leaking into the blood circulation of alcohol-induced animals, further repair hepatic injury, and restore cellular permeability [261,262,263].

The plant extract is believed to have many phytochemicals acting as antioxidants to hinder lipid peroxidation. Around 5.96 mg/g of the leaf extract are composed of phenolic constituents [242]. Based on phytochemical analysis, the water extract of the plant demonstrated greater antioxidant activity than ethanolic extract [264].

Comparing to ethanolic extract, water extract was found to have a higher concentration of flavonoids [265]. At the concentration of 50 mg/kg body weight of albino Wistar rats, the plant extract could restore the anti-oxidative enzymes for hepatoprotection [265]. The plant extract also exhibited free-radical scavenging activity [243]. Lipid peroxide and TBARS in liver could be dropped by 33%–48% after given *Andrographis Herba* extract from 50–200 mg/kg of body weight [237]. During inflammation, lipid peroxides and TBARS appear to be elevated. The surge of lipid peroxidation was reported resulting from the damage of Kupffer cells [238]. The decrease of lipid peroxides and TBARS in the liver of ethanol-induced albino Wistar rats were reported due to the depletion of free-radical generation [266]. Compared to IC_50_ of ascorbate of 410 μg/mL, the free-radical scavenging activity of the plant extract demonstrated IC_50_ of 370 μg/mL. The decrease of lipid peroxides and TBARS in liver reached up to 33%–48% after *Andrographis Herba* extract from 50–200 mg/kg of body weight was administered. On the contrary, 100 mg/kg body weight of silymarin, the synthetic drug, is required to decrease the fatty accumulation in CCl_4_-induced liver inflammatory rat model [267]. Histopathological observation in the rats given herbal extract also demonstrated the obvious drop down in necrosis and fatty degeneration [268].

Hepatic toxicity was revealed by serum activities of bilirubin, alkaline phosphatase, alanine aminotransferase, and aspartate aminotransferase [269,270]. It was also demonstrated that alkaline phosphatase could be the marker of cell membrane functional integrity and cellular leakage. After given *Andrographis Herba* extract at 250 mg/kg body weight for 45 days, these markers demonstrated a decrease of 28%–43%.

The liver protective activity of *Andrographis Herba* was shown to be dose-dependent. The weight of inflamed liver of Swiss male mice was reduced approximately 50% with *Andrographis Herba* with a dosage of 12 mg/kg body weight [238]. Based on liver protein analysis and liver morphology on mice with paracetamol-induced damaged liver, obvious hepatoprotective effect was observed after doses as low as 10 mg/kg of methanolic extract of *Andrographis Herba*.

Nonetheless, andrographolide has a number of bioavailability limits, though it is quickly absorbed into the blood by oral administration. Its elimination half-life increased when in phospholipid-complexed form, thus lowering the clearance of the molecule in this form [271].

#### 5.2.2. *Glycyrrhizae Radix et Rhizoma*

*Glycyrrhizae Radix et Rhizoma*, also known as licorice root, is mostly used to treat HCV and interferon therapy [272]. Its major components include glycyrrhetic acid, β-sitosterol, flavonoids, and hydroxycoumarins. Beta-sitosterol has properties of glucocorticoids and mineralocorticoids. It could decrease alanine transaminase (ALT) level by 20 mg for five days per week for 10-year HCV patients [273], and AST and ALT levels in an animal model of concanavalin A-induced liver damage [274]. Moreover, glycyrrhetic acid could decrease the inflammation response by regulating NF-κB and the MAPK pathway, inhibiting ROS, TNF-α, and pro-inflammatory interleukins like IL-6 and IL-1β [274,275,276]. It improves CCI_4_-induced liver damages, likely by promoting heme oxygenase-1 and down-regulating proinflammatory mediators [277]. It is reported that 18β-glycyrrhetinic acid (GA) down-regulates MyD88 expression and inhibits NF-κB activation, and thus causes reduced macrophage inflammation protein (MIP)-1α expression on Kupffer cells. Overall, GA is involved in anti-inflammation by inhibiting MIP-1α [278]. Diammonium glycyrrhizinate (DG), extract from *Glycyrrhizae Radix et Rhizoma*, can enhance the production of IL-6 and IL-10. DG may exert its hepatoprotection activity by two pathways: inhibiting T-cell-mediated inflammation via an IL-10-dependant pathway, and deterring hepatocytes from apoptosis via an IL-6-dependant pathway [279].

Glycyrrhetic acid was demonstrated to prohibit sialylation of hepatitis B surface antigen (HBsAg), inducing its retention in the trans-Golgi apparatus and regulating glycosylation in a cell culture study [113]. Glycyrrhetic acid is demonstrated its hepatoprotective effect via repressing the activity of prostaglandin E2 production by macrophages and 11-beta-hydroxysteroid dehydrogenase activity, as well as its antioxidative effect via inducing glutathione-*S*-transferases and catalase [280]. Some findings exhibited that the inactivation of NF-κB is associated with an anti-fibrotic effect in the CCl_4_ rat model [281].

In a study of sub-acute liver failure patients administered glycyrrhetic acid daily for a month followed by a two-month glycyrrhetic acid administration every other day, patients were reported to have a better survival rate compared to historical controls from the past decade [282]. Moreover, another study of patients with HCV antibodies treated by glycyrrhetic acid showed an obvious dropping down of relative risk by 2.5-fold in development to HCC [212]. Glycyrrhetic acid treatment also could lower ALT levels but disappeared upon terminating therapy in human trials [283]. The aldosterone-like activities of glycyrrhetic acid result in such adverse effects as hypertension, deterioration of ascites and dropping down of potassium [284].

Pharmacokinetics analysis of glycyrrhetinic acid in humans and experimental demonstrated that glycyrrhetinic acid has a half-life of 3.5 h in humans in the second elimination phase and a biphasic elimination from the central compartment with a dose-dependent second elimination phase [285].

#### 5.2.3. *Ginseng Radix et Rhizoma*

*Ginseng Radix et Rhizoma* is a popular tonic for various diseases such as diabetes and hepatic diseases [286]. The bioactive components of this herb are principally dammarane triterpene *O*-glycosides, in particular, ginsenoside, of which ginsenoside Rd is one of the major active components [287]. Ginsenoside Rd (20-(β-d-glucopyranosyloxy)-12β-hydroxydammar-24-en-3β-yl 2-*O*-β-d-glucopyranosyl-β-d-glucopyranoside) carries diverse bioactivities which are associated with treating metabolic disorders and cancers by its anti-inflammation and immune enhancement activities [288,289]. Ginsenoside Rg 1 (3β,12β-dihydroxydammar-24-ene-6α,20-diyl bis-β-d-glucopyranoside) has demonstrated its ability to block the transcriptional activity of TNF-α-mediated NF-κB, gene expression of COX-2-induced inflammatory enzymes and iNOS [290]. Many studies demonstrated the various pharmacological activities of ginsenosides, including their ability to inhibit inflammation and OS as well as their vasorelaxation effect [291,292,293]. Ginseng regulates antioxidant effects via Nrf2 and levels of antioxidant enzymes by increasing superoxide dismutase and glutathione peroxidase [294,295], and protects rabbit pulmonary endothelium from ROS toxicity [64].

Ginsenoside Rd acts as an antioxidant, as glucose is attached to the sixth carbon instead of the 20th [296]. Ginsenoside Rb1 (20-[(6-*O*-β-d-glucopyranosyl-β-d-glucopyranosyl)oxy]-12β-hydroxydammar-24-en-3β-yl 2-*O*-β-d-glucopyranosyl-β-d-glucopyranoside) showa its protective activities on human umbilical vein endothelial cells [297]. Water extract of Korean red ginseng was demonstrated to promote angiogenesis in human umbilical vein endothelial cells via activating the phosphoinositol-3-kinase (PI3K)/Akt-dependent extracellular signal-regulated kinase 1/2 pathways and endothelial nitric oxide synthase (eNOS) [298].

In clinical studies, the long half-life of ginsenoside Rd, 19.29 h, showed that it might be metabolized moderately after intravenous administration. In rat models, glycosylation and oxygenation were demonstrated to be the main metabolic pathway of Rd in intravenous administration, while deglycosylation was the main metabolic pathway in oral administration [287].

#### 5.2.4. Curcumin

Curcumin, also known as turmeric yellow, and compound of *curcuma longa*, showed various pharmacological effects including anti-inflammatory, anti-oxidative, and hepatoprotective activities [299]. It was reported to decrease the production of cytokines including TNF-α and TNF-β via inhibiting NF-κB, and thus it likely possesses the prophylactic effect on liver diseases by anti-inflammatory effects [300]. It could decrease hepatic MDA and inhibit NF-κB activation in alcohol-induced female Sprague-Dawley rats [146]. It also demonstrated its anti-oxidant effect via inhibiting ROS generation in ethanol-exposed mice. However, it is not suggested as a favorable treatment option due to its low bioavailability and rapid metabolism.

Moreover, curcumin acts on liver injuries by targeting multiple sites, for example platelet-derived growth factor-β receptor (PDGF-βR) [301], tissue growth factor β (TGFβ) [302,303], toll-like receptors (TLRs) [304], matrix metalloproteinases (MMPs) [301,305], peroxisome proliferator-activated receptors (PPARc) [305], apoptotic pathway [303,306] microRNAs [307], and inflammatory cytokines [304,305,308,309].

In an *in vitro* study, curcumin was also demonstrated to inhibit the stimulatory effects of leptin by suppressing the phosphorylation and expression of leptin receptor (Ob-R) [310]. The latter is initiated by decreasing OS and stimulating PPARc activity [305]. Moreover, it abolished stimulatory effects of leptin on HSC activation through regulating intracellular lipids and elevating AMPK activity in HSCs. Curcumin inhibits HSC activation by preventing leptin from increasing intracellular glucose levels in activated HSCs. Also, curcumin inhibits HSC activation by activating AMPK activity, leading to the induction of gene expression associated with elevating triglycerides (TGs) and intracellular fatty acids (FAs), and accumulating lipids [305]. It is also found to inhibit HSC activation by stopping AGE-caused activation of leptin signaling in activated HSC [311]. Moreover, its activation to AMPK showed various functions in other cell types, such as 3T3-L1 adipocytes [312], HT-29 colon cancer cells [311] and hepatoma cells [311]. These findings postulated that curcumin possibly exerts specific activities on lipid accumulation according to cell types and on regulating gene expression, in which curcumin exhibits its epigenetic events.

Interestingly, curcumin at lower concentrations acts as a powerful agent in modulating miRNAs expression, particularly inactivating or activating gene expression, via exerting its effect on HDACs and acetyl transferases [313].

However, the kinetic behavior of curcumin degradation is complicated as its half-life varies among different pH and solvents. The half-life of curcumin is around 6.6 × 10^3^ h at pH 1.23, and shortened when the pH is elevated to 7.98; the stability of curcumin followed the decreasing trend: methanol (92.7 h) > ethyl acetate (15.1 h) > acetonitrile (6.3 h) > chloroform (2.7 h) [314].

#### 5.2.5. *Lycii Fructus*

*Lycii Fructus,* also known as Wolfberry, the fruit of plant *Lycium barvarum* of the family Solanaceae, is a popular herbal drug targeting liver and eyes [315]. *Lycii Fructus* carry amino acids, betaine, flavonoids, scopoletin (6-methoxy-7-hydroxycoumarin, also known as, scopoletol, gelseminic acid, ecopoletin, and chrysatropic acid), cerebroside, minerals, β-sitosterol, vitamins (e.g., ascorbic acid, thiamin and riboflavin), stable vitamin C analog 2-*O*-β-d-glucopyranosyl-l-ascorbic acid, carotenoids (β-carotene and zeaxanthin), glucosylated precursor, and plentiful polysaccharides (LBPs), which can be found in 5%–8% of dried fruits [316]. LBPs are regarded as the most crucial components in *Lycii Fructus* and associated with various effects, most of which depend on galacturonic acid, and the bioactivities are often reversely proportional to molecular weights. LBPs have been demonstrated to be effective in promoting health, and therapies for different diseases in clinical and preclinical studies.

For example, a zebra fish model showed positive effects of LBPs on a p53-mediated signaling pathway and cell apoptosis, which may be responsible for aging. Its mechanism was shown by SA-β-gal and phenotypic assays and evaluated by survival rates *in vivo* [317].

Moreover, LBPs were demonstrated to have promising effects against OS and stimulate immune functions in an aged mice model study [318]. By measuring total antioxidant capacity (TAOC), superoxide dismutase (SOD), catalase (CAT), and glutathione peroxidase (GPx) of aged mice treated with LBPs, elevated antioxidant effects and alleviated endogenous lipid peroxidation were observed in the brain, liver, lungs and heart. The elevated non-enzymatic system and antioxidant enzymes may be one of the mechanisms of the lowering effect on lipid peroxidation. The immune functions of aged mice treated with LBPs was also restored to normal as evaluated by phagocytic index, phagocytic activity, as well as spleen and thymus index. Moreover, the MDA level and lipofuscin level (a key indicator for oxidative injury), which were obviously higher in aged mice, was suppressed by LBP administration [318].

Another study of mice demonstrated that administration of LBPs dose-dependently significantly elevated peripheral and hepatic antioxidant enzymes activities (CAT, SOD, GPx, and TAOC level) and GSH level, but dropped down MDA and NO-level [319].

Clinical study showed different effects on apoptosis in human hepatic cancer SMMC-7721 cells, cell cycle distribution, and proliferation with different amounts of LBPs at doses of 50–400 mg/L for two, four and six days. The proliferation of human hepatoma QGY7703 cells was suppressed by 100 mg/L LBPs, hence leading to cell cycle arrest, and significantly elevated intracellular Ca^2+^ level [320]. Another *in vivo* study of 50 Chinese healthy adults demonstrated that GPx and serum SOD level were elevated by 8.7% and 8.4% respectively via administration of 13.6 mg/mL LBPs at a dose of 120 mL/day [321].

#### 5.2.6. *Coptidis Rhizoma*

Berberine (BBR), an alkaloid isolated from *Coptidis Rhizoma*, showed its anti-steatotic effect via reactivating AMPK and up-regulating low-density lipoprotein receptor expression by the extracellular signal-regulated kinase (ERK) pathways and c-Jun N-terminal kinase (JNK) [322,323]. Moreover, BBR also demonstrated its ability to reduce hepatic inflammatory response, by the modulation of the NF-κB signaling pathway [324,325].

BBR showed obvious inhibitory effects on OS in a series of diabetic animal models [326,327,328,329,330,331,332,333,334] and cells cultured with high glucose-containing medium [141]. The antioxidant activity of BBR was demonstrated by changing antioxidant enzymes and OS markers including malondialdehyde (MDA), a product of lipid peroxidation which increased during OS [335], and glutathione (GSH), which often declines during OS [336]. Antioxidant enzymes are a part of the antioxidant defense mechanisms, which are responsible for keeping the balance of redox in organisms and could be damaged in the pathogenesis of diabetes mellitus [337].

According to both *in vitro* and *in vivo* studies, the anti-inflammatory activity of BBR was observed by decreasing the pro-inflammatory cytokines and acute phase proteins [326,327,338,339,340,341,342,343]. In pancreatic β-cells, cultured metabolic cells (adipocytes and liver cells), or immunocytes (macrophages and splenocytes), BBR prohibited the production of C-reaction protein (CRP) and haptoglobin (HP), matrix metalloprotease 9 (MMP9), inducible nitric oxide synthase (iNOS), cyclooxygenase-2 (COX2), TNF-α, IL-6, IL-1β, and monocyte chemoattractant protein 1 (MCP-1) [338,339,340,341,344]. The anti-inflammatory activity of BBR demonstrated in insulin resistant HepG2 cells was related to its insulin-sensitizing effect [339]. BBR administration obviously lowered cytokine production and serine phosphorylation, whereas it elevated insulin-mediated tyrosine phosphorylation of IRS in HepG2 cells treated with palmitate [339].

### 5.3. Composite Formulae

#### 5.3.1. Xiao-Cha-Hu-Tang

Xiao-Cha-Hu-Tang, also known as Sho-saiko-to in Japan, had been used for treating liver diseases since ancient China, and comprises seven herbal constituents including: pinellia tuber, ginger rhizome, glycyrrhiza root, bupleurum root, jujube fruit, scutellaria toot, and ginseng root. It has been demonstrated as an effective anti-inflammatory agent via reducing inflammatory process and regulating ALT levels [325]. Saikosaponin-A (SSA) and Saikosaponin-D (SSD) are two extracts from bupleurum, one of the constituents of Xiao-Cha-Hu-Tang. SSA, an antioxidant, can increase anti-inflammatory cytokine IL-10, inhibit hepatic proinflammatory cytokines, such as IL-1β, IL-6 and TNF-α, as well as suppress inflammation and fibrogenesis [308]. The formula has shown its efficacy for chronic hepatitis and liver cirrhosis.

#### 5.3.2. Shi-Quan-Da-Bu-Tang

This formula, also known as Juzen-taiho-to in Japan, is a famous tonic remedy and has been used for treating general weakness, anemia, anorexia, and fatigue for nearly a thousand years in China. It consists of 10 herbal components including Panax ginseng, *Atractylodes macrocephala*, *Angelica sinensis*, *Cinnamomum cassia*, *Paeonia lactiflora*, *Astragalus membranaceus*, *Poria cocos, Rehmannia glutinosa*, *Liqusticum wallichii*, and *Glycyrrhiza uralensis*.

It was demonstrated to inhibit the secretion of IL-2, but promote IL-4, IL-5, IL-6 and INF-γ from stimulated hepatic lymphocytes. The amount of CD3-positive intermediate cells among NKT cells was elevated after oral intake of the formula. It increased IL-12 mRNA transcription in the liver, which may also result in the induction of NKT cells [345]. Hence, this formula can suppress hepatic inflammation and induce NKT cells. However, some studies pointed out that the formula could not improve liver dysfunction, as its pre-surgical administration appeared to inhibit the post-surgical hyperammonemia, but did not improve post-surgical liver dysfunction [346].

Xiao-Cha-Hu-Tang and Shi-Quan-Da-Bu-Tang both demonstrated inhibition of fibrosis and necroinflammation in the livers of a murine NASH model, though the mechanisms were not clear [347]. Overall, they both appeared to be effective anti-inflammation agents by inducing NKT cells. However, there are some cases of adverse events and hepatotoxicity resulting from herbal medicines [348]; it was observed that Xiao Chaihu Tang (Sho-saiko-to) may cause acute interstitial pneumonia in chronic hepatitis patients, when used alone or in combination with interferon [349].

## 6. Conclusions and Future Perspectives

Although there are some negative opinions [348,349,350,351], evidence shows that CMHs are effective anti-inflammatory and anti-oxidative agents with milder and fewer side effects, and can act as tonics to prevent diseases in prophylactic strategies. 

In the theory of TCM, herbal composite formulae are composed of several kinds of herbs, based on the syndrome differentiation according to patient symptoms. Therefore, it is relatively difficult to probe which component from the formula is the main contributor to the therapy and without applying the syndrome differentiation, the results of most of the current randomized clinical trials for CMHs are difficult to assess. This may explain the less favorable effects of CHMs in treating and preventing hepatic diseases reported in clinical trials, though desirable effects are obtained from laboratory experiments.

Actually, in the clinical practice of TCM, herbs do not work independently, but are prescribed in formulae. It is believed that these anti-oxidative and anti-inflammatory activities of TCM may stem from its additive or synergistic active effects. Therefore, large-scale clinical studies based on TCM syndrome differentiation should be performed to further evaluate the anti-oxidative and anti-inflammatory effects of CMHs on liver diseases.

## Abbreviations

AC	Autoimmune cholestatic liver diseases
ACC	Acetyl-CoA carboxylase
ACLY	ATP-citrate lyase
ADH	Alcohol dehydrogenase
AIH	Autoimmune hepatitis
ALD	Alcoholic liver disease
ALP	Alkaline phosphatase
ALT	Alanine transaminase
ALDH	Aldehyde dehydrogenases
AMPK	AMP-activated protein kinase
AOX	Acyl-CoA oxidase
ARE	Antioxidant response element
AST	Aspartate aminotransferase
BHA	Butylated hydroxyanisole
bw	Body weight
CAT	Catalase
CCl4	Carbon tetrachloride
CMHs	Chinese Medicinal Herbs
COX-2	Cyclooxygenase-2
CPT-1	Carnitine palmitoyltransferase-1
CYP2E1	Cytochrome P450 2E
DGAT	Diacylglycerol acyltransferase
EGCG	Epigallocatechin-3-gallate
ER	Endoplasmic reticulum
FAS	Fatty acid synthase
GGT	γ-Glutamyl transferase
GPX	Glutathione peroxidase
GSH-Px	Glutathione peroxidase
GSH	Glutathione
GRD	Glutathione reductase
GST	Glutathione S-transferase
HDL	High density lipoprotein
HCV	Hepatitis C virus
IL-6	Interleukin 6
INH	Anti-tuberculosis agent isoniazid
iNOS	Inducible nitric oxide synthase (iNOS)
INrf2	Inhibitor of Nrf2
IKK β	IkB kinase-β
IRS	Insulin receptor substrate
JNK	C-Jun N-terminal kinases
Keap1	Kelch-like ECH-associated protein-1
LBP	LPS-binding protein
LPO	Lipid peroxidation
LPS	Lipopolysaccharide
LDH	Lactate dehydrogenase
LDL	Low density lipoprotein
MCAD	Mitochondrial medium-chain acyl-CoA dehydrogenase
MCP-1	Monocyte chemotactic protein-1
MDA	Malondialdehyde
MEOS	Microsomal ethanol oxidizing system
MyD88	Myeloid differentiation factor 88
NADPH	Nicotinamide adenine dinucleotide phosphate-oxidase
NAFLD	Non-alcoholic fatty liver disease NAFLD
NF-κB	Nuclear factor-κB
NO	Nitric Oxide
NQO1	NAD(P)H Dehydrogenase, Quinone 1
Nrf1	Nuclear respiratory factor 1
Nrf2	Erythroid 2-related factor 2
PGC-1α	Peroxisome proliferator-activated receptor g coactivator α
PKC	Protein kinase C
PPARα	Peroxisome proliferator activated receptor RNS Reactive nitrogen species
ROS	Reactive oxygen species
SCD-1	Stearyl CoA desaturase-1
SIRT1	Sirtuin 1
SOD	Superoxide dismutases
SREBP-1c	Sterol regulatory element-binding protein-1c
STAT-3	Signal transducer and activator of transcription-3
TAA	Thioacetamide
TB	Total bilirubin
TBARS	Thiobarbituric acid-reactive substances
TC	Total cholesterolsTCM Traditional Chinese Medicine
TG	Triglyceride
TLR4	Toll-like receptor 4
TNF	Tumor necrosis factor
TNF-α	Tumor necrosis factor-α
TRIF	TIR-domain-containing adapter-inducing interferon-b
ZO-1	Zonula occludens-1
